# Psychological assessment and support in functional neurological disorder: a longitudinal study

**DOI:** 10.3389/fpsyg.2025.1506069

**Published:** 2025-02-10

**Authors:** Yvonne Radin, Matteo Bulfon, Paola Caruso, Egidio Barbi, Paolo Manganotti, Andrea Clarici

**Affiliations:** ^1^Institute for Maternal and Child Health IRCCS Burlo Garofolo, Trieste, Italy; ^2^Department of Medical, Surgical and Health Sciences, Cattinara Hospital, University of Trieste, Trieste, Italy

**Keywords:** functional neurological disorder, Affective Neuroscience Personality Scales, psychoanalysis, PANIC system, SWAP-200, Stroke Mimic

## Abstract

In the Department of Neuroscience in Clinical Neurological unit of our hospital, between 2020 and 2024, 58 adult patients were diagnosed with functional neurological disorder (FND). Out of these, 42 patients agreed to participate in a structured intervention consisting of 10 sessions of psychotherapy. This study aimed to investigate the demographic and clinical characteristics of the patient cohort, examine their reported symptoms, and evaluate the effectiveness and adherence to the psychotherapy program. The Affective Neuroscience Personality Scales (ANPS) were administered to assess the affective states of the patients, while the Shedler-Westen Assessment Procedure (SWAP-200) was used to evaluate their main personality style. A central objective of the research was to explore patterns or correlations between self-reported data from the patients and the evaluations completed by their therapists. This comparison sought to identify any alignment or discrepancies in the perception of symptoms and therapeutic progress, as measured by both the ANPS and SWAP-200 scales. The study’s preliminary findings are reported to provide valuable insights into the impact of psychotherapeutic interventions for FND, including an understanding of the degree to which patient self-reports correspond with clinical assessments. These results will inform the optimization of treatment strategies and enhance patient outcomes by integrating patient feedback with clinical evaluations. The research contributes to the broader knowledge of FND management, emphasizing the importance of aligning patient and therapist perspectives in the therapeutic process.

## Introduction

Functional neurological disorders (FND) represent an emerging pathological condition characterized by neurological symptoms that lack a clear organic cause, often manifesting as sensory-motor disturbances. Focusing on improving quality of life (QoL) has emerged as a critical goal in managing FND, aligning with integrative therapeutic approaches (Myers et al., 2021). These disorders are indeed frequently associated with impairing conditions like chronic fatigue syndrome and fibromyalgia, posing significant challenges in understanding their aetiology and management (Stone et al., 2020). FNDs can cause significant distress and disability, representing a critical condition requiring prompt diagnosis and accurate follow-up to prevent acute recurrences and misdiagnosis (Ludwig et al., 2018; Lidstone et al., 2020). Emerging models suggest that FND may stem from abnormalities in Bayesian inference processes in the brain, where excessive precision is attributed to *a priori* predictions, disrupting sensory and attentional processing (Edwards et al., 2012). This Bayesian perspective provides a framework for understanding the variability in symptoms, the complexities in diagnosis, and the challenges in treatment, situating FND within Friston’s predictive coding and “free energy principle” (Friston, 2010).

The “free energy principle” posits that the brain functions as an inference machine, continually updating its predictions to minimize surprise or prediction error (Parr and Friston, 2019), thereby constructing a generative model of its environment. Dysregulations in this process can manifest FND symptoms, mainly when emotional conflicts drive an excessive focus on specific predictions, resulting in misinterpretations of bodily sensations (Solms and Friston, 2018). Understanding these processes requires examining the role of primary emotional systems, as Panksepp (Panksepp, 2010) identified, which include SEEKING, PLAY, CARE, LUST, FEAR, ANGER, and PANIC/GRIEF. Dysfunctions in these systems, potentially involving alterations in dopaminergic and opioidergic pathways, may contribute to the sensory, motor, and emotional symptoms seen in FND (Panksepp and Watt, 2011).

Recent findings suggest that psychotherapy could be a valuable tool to aid FND patients. For instance, Cognitive Behavioural Therapy (CBT) and Psychodynamic Therapy (PDT) could offer medium-sized benefits for physical symptoms, mental health, and QoL. CBT targets maladaptive beliefs and avoidance behaviours, while PDT addresses emotional and interpersonal conflicts contributing to symptom maintenance (Gutkin et al., 2020).

This study aims to explore the psycho-emotional structure of FND patients (who accepted a psychotherapeutic intervention) using the Affective Neuroscience Personality Scales (ANPS) and the Shedler-Westen Assessment Procedure (SWAP-200).

The hypothesis is that therapy will improve patients’ ability to manage symptoms (Sandler and Joffe, 1968; Fonagy, 2005) and enhance their understanding of triggers, with particular focus on the role of attentional dynamics in functional neurological disorder (Edwards et al., 2012). However, given the exploratory nature of the study and the lack of prior research, it is difficult to formulate precise hypotheses regarding the ANPS results.

The study’s limitations include the absence of a control group and the small, heterogeneous sample, which may affect the generalizability of the findings.

## Materials and methods

The study was conducted at Cattinara Hospital, a tertiary care center located in Trieste, Italy, which includes a specialized Neurological Clinic offering comprehensive diagnostic and therapeutic services for a wide range of neurological disorders.

Patients were initially enrolled from the Emergency Department of Cattinara Hospital after presenting with acute neurological symptoms from January 1st 2018 to December 31th 2023. Following initial stabilization and assessment, they were referred to the Neurological Clinic for further evaluation. For 58 out of 128 patients the diagnosis was confirmed. For these 58 patients, clinical and demographic characteristics were collected (Table 1). Before their involvement in the study, informed consent was obtained from all participants, and ethical considerations were strictly adhered to throughout the research process. The study sample consists of 42 patients who accepted a psychotherapeutic intervention out of the initial 58 individual diagnosed with FND.

**Table 1:** Demographic data and medical history of the examined sample.

Variable	Stroke Mimic (*N* = 18)	Movement (*N* = 29)	PNES (*N* = 11)	TOTAL (*N* = 58)
Demographics characteristics				
Sex (Male)	6 (33.3%)	4 (13,8%)	0 (0.0%)	10 (17.2%)
Age at the onset	51.8 ± 15.8	49.4 ± 15.4	43.1 ± 16.0	49.0 ± 15.3
Education, years	11.83 ± 2.01	13.14 ± 3.51	14.03 ± 2.16	12.93 ± 2.98
Accepted psychological support (yes)	14 (77.8%)	20 (69.0%)	8 (72.7%)	42 (72.4%)
Clinical characteristics				
Psychiatric disorders				
Anxiety	7 (29.2%)	11 (45.8%)	6 (25.0%)	24 (100%)
Panic Attack	4 (23.5%)	8 (47.1%)	5 (29.4%)	17 (100%)
Depression	5 (45.4%)	5 (45.4%)	1 (9.1%)	11 (100%)
Acute Stress	6 (54.5%)	4 (36.4%)	1 (9.1%)	11 (100%)
Other	1 (20.0%)	1 (20.0%)	3 (60.0%)	5 (100%)
Not Reported	6 (35.2%)	9 (53.0%)	2 (11.8%)	17 (100%)
Remote medical history				
Orthopaedic conditions	13 (41.9%)	18 (58.1%)	0 (0.0%)	31 (100%)
Cardiovascular conditions	13 (59.1%)	6 (27.3%)	3 (13.6%)	22 (100%)
Neurological conditions	6 (33.3%)	9 (50.0%)	3 (16.7%)	18 (100%)
General surgical interventions	7 (38.9%)	10 (55.6%)	1 (5.6%)	18 (100%)
Endocrine and metabolic conditions	9 (50.0%)	7 (38.9%)	2 (11.1%)	18 (100%)
Gastrointestinal conditions	5 (50.0%)	3 (30.0%)	2 (20.0%)	10 (100%)
Neoplasms	4 (44.4%)	3 (33.3%)	2 (22.2%)	9 (100%)
Ophthalmic conditions	5 (62.5%)	2 (25.0%)	1 (12.5%)	8 (100%)
Rheumatological conditions	3 (37.5%)	3 (37.5%)	2 (25.0%)	8 (100%)
Respiratory conditions	3 (42.9%)	3 (42.9%)	1 (14.3%)	7 (100%)
Gynaecological/obstetrical conditions	4 (66.7%)	2 (33.3%)	0 (0.0%)	6 (100%)
Dermatological conditions	2 (100%)	0 (0.0%)	0 (0.0%)	2 (100%)
Not relevant	1 (9.1%)	4 (36.4%)	6 (54.5%)	11 (100%)
Neurological examination sings				
Motor disturbances/deficits	6 (46.2%)	6 (46.2%)	1 (7.7%)	13 (100%)
Involuntary movements	0 (0.0%)	9 (100%)	0 (0.0%)	9 (100%)
Sensory disturbances	4 (50.0%)	5 (55.6%)	1 (14.3%)	10 (100%)
Gait/balance disturbances	2 (28.6%)	5 (71.4%)	0 (0.0%)	7 (100%)
Cognitive/ consciousness disturbances	2 (50.0%)	0 (0.0%)	2 (50.0%)	4 (100%)
Negative	8 (27.6%)	12 (41.4%)	9 (31.0%)	29 (100%)

Values are presented as *M* (SD) for continuous variables and *n* (%) for categorical variables.

Among the 42 patients, seven dropped out before completing the therapy sessions (16.7%). Additionally, there was missing data for the assessments, with four patients (9.5%) not completing the Affective Neuroscience Personality Scales (ANPS) and four patients (9.5%) missing the SWAP-200 assessment because of a lack of information emerged during the psychotherapy sessions. The psychological support provided was based on two theoretical cornerstones: the fundamental importance of the meaning of the symptom, i.e., the intimate connection between affective activation and adaptive reactions (Solms and Friston, 2018; Solms, 2021) and Kernberg’s Transference-Focused Psychotherapy (TFP) approach (Kernberg and Caligor, 2005; Kernberg, 1984) which focuses on understanding and resolving unconscious conflicts by exploring the transference relationship—the dynamic between the patient and therapist as a reflection of the patient’s internalized relationships and emotional struggles. The intervention was delivered in 10 weekly sessions of 1 h each. Patients were randomly assigned to one of four psychotherapists, all with a psychoanalytic orientation. Randomization ensured an unbiased distribution of participant characteristics, minimizing confounding factors and enabling the assessment of both the overall intervention effectiveness and potential therapist-specific effects.

Twenty-seven total patients successfully completed the clinical trial, including the testing part. To assess the psychological dimensions of FND and the possible impact of the treatment, we employed the Affective Neuroscience Personality Scales (ANPS) and the Shedler-Westen Assessment Procedure (SWAP-200). These tools were specifically chosen for their complementary strengths in evaluating emotional dysregulation and personality constructs that are hypothesized to underlie FND symptomatology.

The ANPS (Davis et al., 2003) was selected based on the theoretical framework proposed by Panksepp, to provide insights into potential affective imbalances that may contribute to the manifestation of FND symptoms.

The SWAP-200 (Westen and Shedler, 2007), on the other hand, offers a unique advantage as it is completed by therapists rather than the patients themselves. This feature allows for an independent, expert-driven assessment of a broad range of personality traits and disorders (The Pandas Development Team, 2023). Moreover, it enables the identification of consistent personality characteristics that may either correlate with the ANPS findings or emerge as common themes across therapist observations.

By integrating data from both tools, we aimed to achieve a nuanced understanding of the emotional and personality dimensions of FND, exploring potential interrelations and validating therapist-perceived patterns with empirical measures of affective systems.

After collecting ANPS result in the three phases (T0, T1, T2) we compared T0 results against Italian normative data. However, our sample consists of mostly females (23 females and 4 males), leading to possible distortions. As such, we performed a Welch’s t-test on the female-only sample who had completed the ANPS test prior to the start of the interviews (T0), to assess whether significant differences existed between our female patient group and the female normative data (Pascazio et al., 2015). This statistical test was chosen for its robustness in handling unequal sample sizes and variances. For the subsequent analysis, both males and females will be considered.

Afterwards, a repeated measures ANOVA was conducted considering time as the factor within subjects using Jamovi software (Jamovi Project, 2022) to evaluate changes in the ANPS scores over time (*N* = 27). The ANPS was administered at three distinct time points: Baseline (before the initiation of therapy), Midpoint (at the end of the psychotherapy sessions), and Follow-up (2 months after the end). The assumption of sphericity was tested using Mauchly’s test to ensure the validity of the ANOVA results. The sphericity test was non-significant (*W* = 0.844 *p* = 0.120), indicating that the assumption of sphericity was met. As such, no correction for degrees of freedom was necessary.

In addition to the ANPS score analysis, correlation coefficients were calculated to explore the relationships between the PD-T SWAP-200 profiles and the ANPS variables, while to explore the relationships between the Q-T SWAP-200 factors and the ANPS variables. Q-T (Quantitative Trait) measures the severity of personality pathology on a spectrum, representing the overall maladaptive functioning level across domains of personality traits.

PD-T (Personality Disorder Types) Classifies personality characteristics into specific personality disorder prototypes, aligning patients with empirically derived personality styles and disorders, such as borderline, narcissistic, or obsessive-compulsive types.

To assess the normality of the distribution for each of the 30 variables in the dataset about ANPS and SWAP-200, the Shapiro–Wilk test was conducted revealing that 25 of the 30 variables in the dataset were normally distributed, as evidenced by *p*-values greater than 0.05. However, five variables exhibited *p*-values below the 0.05 threshold, indicating that these variables do not follow a normal distribution. Specifically: PD-T Narcissistic (*p*-value: 0.033), Q-T Antisocial (*p*-value: 0.042), Q-T Narcissistic (*p*-value: 0.002), Q-T Depressive high function (*p*-value: 0.034), Q-T Hostile (*p*-value: 0.009). These findings suggest that these five variables significantly deviate from normality, which may influence the statistical methods used in subsequent analyses. Given that, we have chosen Pearson’s correlation for the variables that conform to normal distribution. For the pairings where one of the variables did not follow a normal distribution, we used instead Spearman’s correlation.

We used Spearman’s correlation (the explanation about this choice is in the result section) the SWAP-200, which provides a detailed assessment of personality traits and potential psychiatric disorders, was analysed to determine how these profiles correlated with the emotional systems measured by the ANPS. These correlations were calculated using Python, with the pandas and numpy libraries used for data manipulation, and the seaborn library employed for visualization (The Pandas Development Team, 2023).

**Figure 1:**
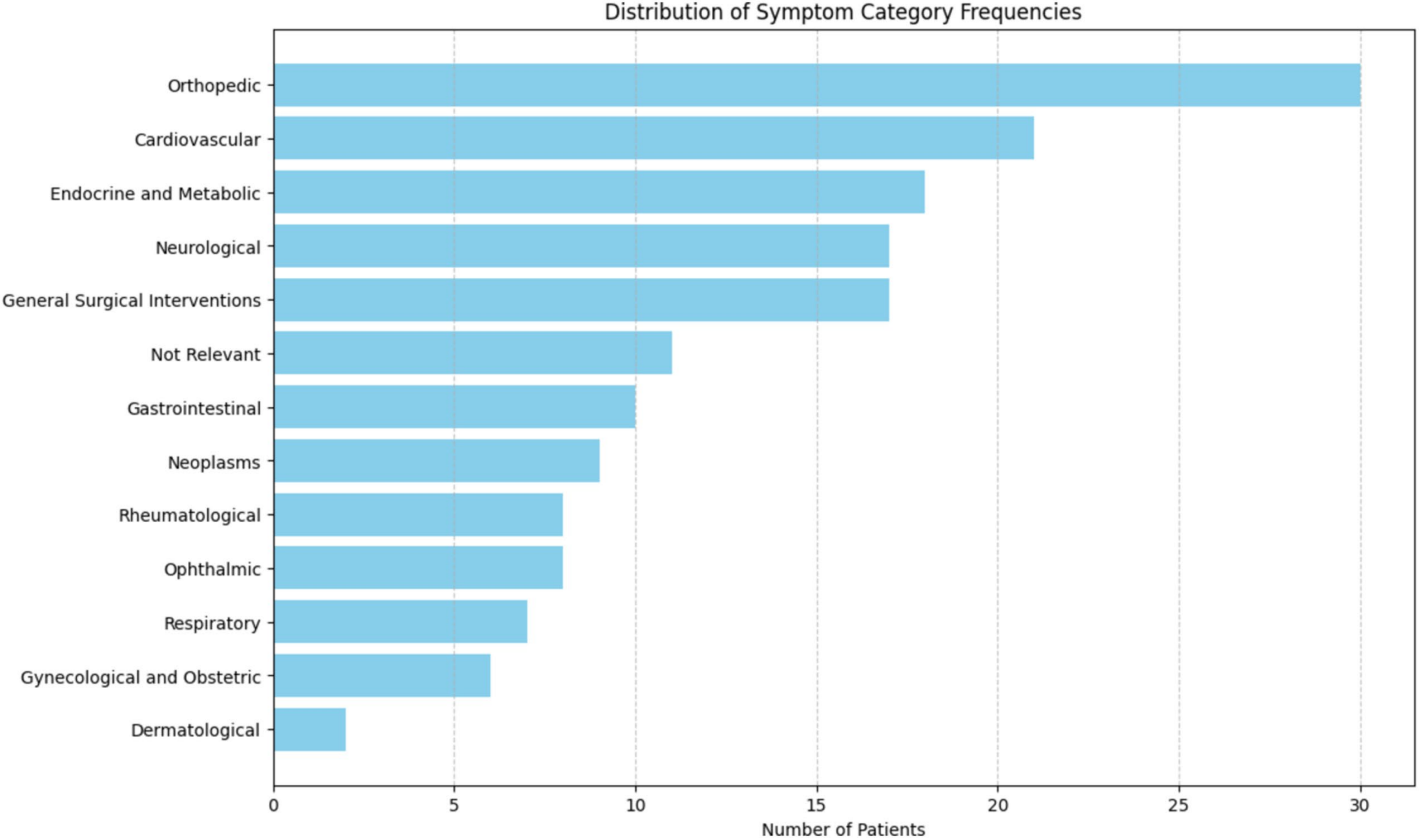
This bar chart displays the distribution of remote medical history among the patients, sorted from the most to the least frequent. The horizontal bars represent the number of patients affected by each category, with the highest frequencies at the top.

The results of these statistical analyses were interpreted with a focus on clinical relevance, emphasizing how changes in ANPS scores over time and the correlations between SWAP-200 profiles and ANPS variables might inform the understanding and treatment of FND. The effect size calculations were reported alongside 95% confidence intervals to provide context for the magnitude of the observed effects (Figure 1).

## Results

Table 1 summarizes clinical and demographic characteristics of all the 58 patients that were considered for the study. Three diagnostic groups were identified: Stroke Mimic (*N* = 18), Movement Disorders (*N* = 29), and Psychogenic Nonepileptic Seizures (PNES, *N* = 11). The first group includes patients with symptoms that mimic a stroke, such as hemiparesis and language disturbances (Caruso et al., 2024). The second group refers to patients showing motor impairments such as tremors and dystonia (Serranová et al., 2023). The third one pertains to patients presenting a wide range of symptoms mimicking epileptic seizures (Bompaire et al., 2021). The participants’ mean age (calculated as the age each patient had at the first access to the ER for functional symptoms) varied slightly among the groups, with the Stroke Mimic group having an average onset age of 51.8 years (SD = 15.8), while the Movement Disorders and PNES groups had average ages of 49.4 (SD = 15.4) and 43.1 years (SD = 16.0) respectively. Education levels were slightly higher in the PNES group (*M* = 14.03, SD = 2.16) compared to the Stroke Mimic (*M* = 11.83, SD = 2.01) and Movement Disorders (*M* = 13.14, SD = 3.51) groups. Most participants across all groups accepted psychological support, with the highest proportion in the Stroke Mimic group (77.8%).

The prevalence of psychiatric disorders varied across the groups, with Anxiety being the most common in the Movement Disorders group (45.8%), followed by the Stroke Mimic (29.2%) and PNES (25.0%) groups. Panic attacks were reported most frequently in the Movement Disorders group (47.1%). Depression and Acute Stress were more evenly distributed, with slight variations among the groups. Cardiovascular conditions were more common in the Stroke Mimic group (59.1%), while the Movement Disorders group had a higher prevalence of neurological and surgical interventions. In the context of Neurological Examination Signs Motor disturbances/deficits were reported equally in the Stroke Mimic and Movement Disorders groups (46.2% each), while sensory disturbances were more common in the Movement Disorders group (55.6%). Involuntary movements were exclusively reported in the Movement Disorders group (100%), and cognitive/consciousness disturbances were evenly split between the Stroke Mimic and PNES groups. Overall, 72.4% of the total sample accepted psychological support, reflecting a relatively high acceptance rate across all diagnostic categories. Notably, there were only six dropouts from the therapy sessions, and unfortunately, one participant passed away during the study (Table 2).

**Table 2:** Variable: ANPS and SWAP-200 completed (*N* = 27).

SWAP PD-T factors		SWAP QT factors		ANPS variables	
Paranoid	42.45 ± 7.02	Antisocial	41.86 ± 4.78	SEEKING	24.74 ± 6.25
Schizoid	47.71 ± 6.21	Schizoid	47.37 ± 7.04	PLAY	23.30 ± 6.00
Schizotypic	47.29 ± 7.25	Paranoid	48.44 ± 6.28	CARE	29.30 ± 5.78
Antisocial	42.29 ± 4.53	Obsessive	52.02 ± 8.77	FEAR	24.74 ± 6.81
Borderline	47.80 ± 7.73	Istrionic	52.75 ± 5.89	ANGER	18.74 ± 8.21
Istrionic	47.30 ± 6.65	Narcissistic	37.44 ± 7.09	SADNESS	24.93 ± 6.39
Narcissistic	41.19 ± 6.55	Avoidant	48.87 ± 5.05	LUST	24.07 ± 7.82
Avoidant	48.42 ± 5.29	Depressive, High funct.	56.71 ± 7.44		
Dependent	51.54 ± 6.46	Emotional Disreg.	50.46 ± 9.21		
Obsessive	47.71 ± 4.99	Dependent	47.51 ± 6.93		
High Funct.	55.38 ± 8.92	Hostile	42.76 ± 7.91		
		High Funct.	55.38 ± 8.92		

**Figure 2:**
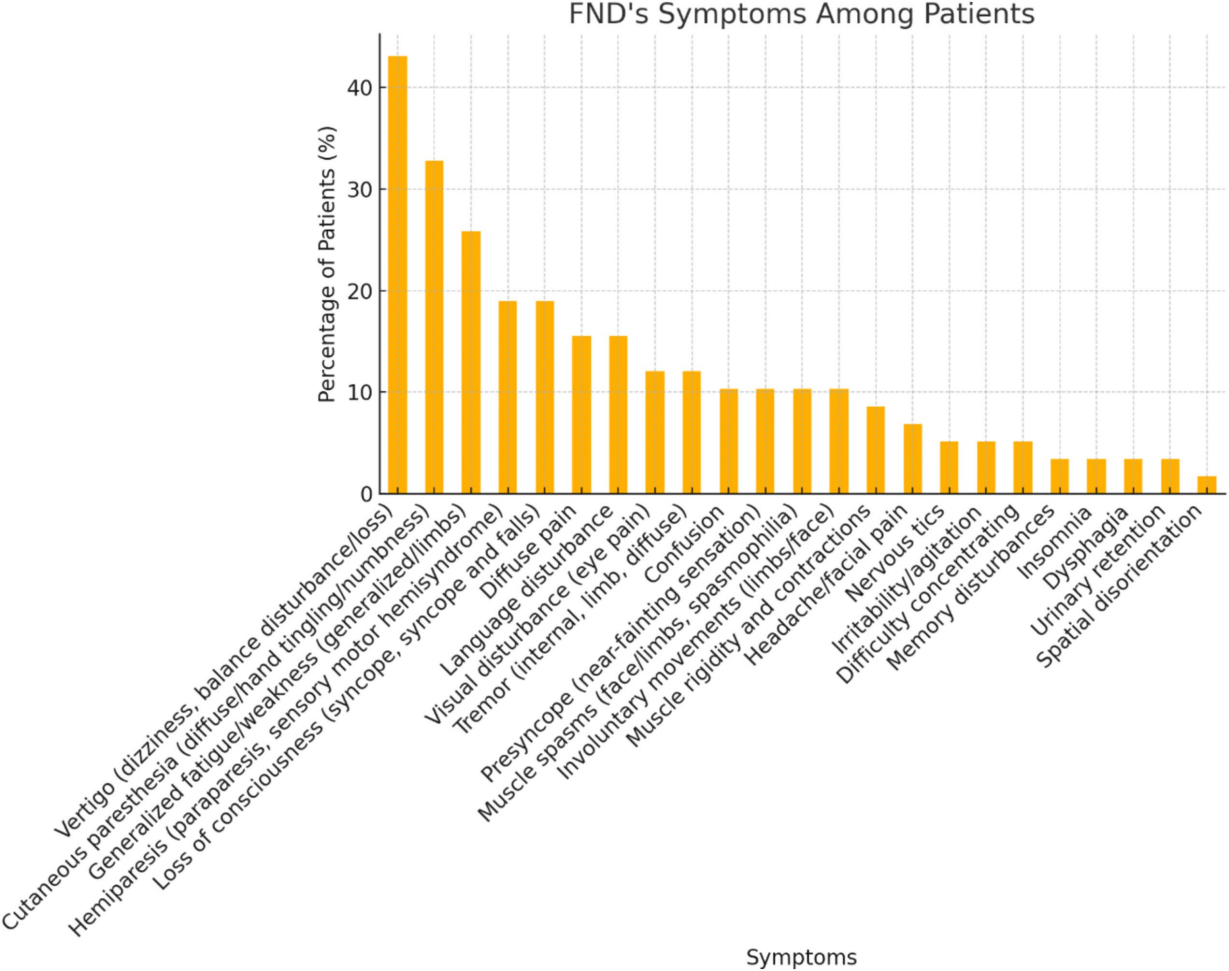
This chart illustrates the distribution of symptoms among patients diagnosed with functional neurological disorder (FND). Each bar represents the percentage of patients exhibiting a specific symptom.

The bar chart in Figure 2 illustrates the distribution of reported functional symptoms among the patient cohort, highlighting the most prevalent symptoms associated with functional neurological disorders. Vertigo (Dizziness/Balance Disturbance) is the most reported symptom, affecting 43.1% of the cohort with a chi-square value of 44.78 (*p* < 0.001), significantly more frequent than expected.

Cutaneous Paresthesia is the second most common symptom, representing 32.8% of the population, and with a chi-square value of 19.76 (*p* < 0.001). Generalized Fatigue/Weakness (Asthenia) is reported by 15 patients, which is 25.9% of the cohort (chi-square = 8.69, *p* = 0.0032).

**Figure 3:**
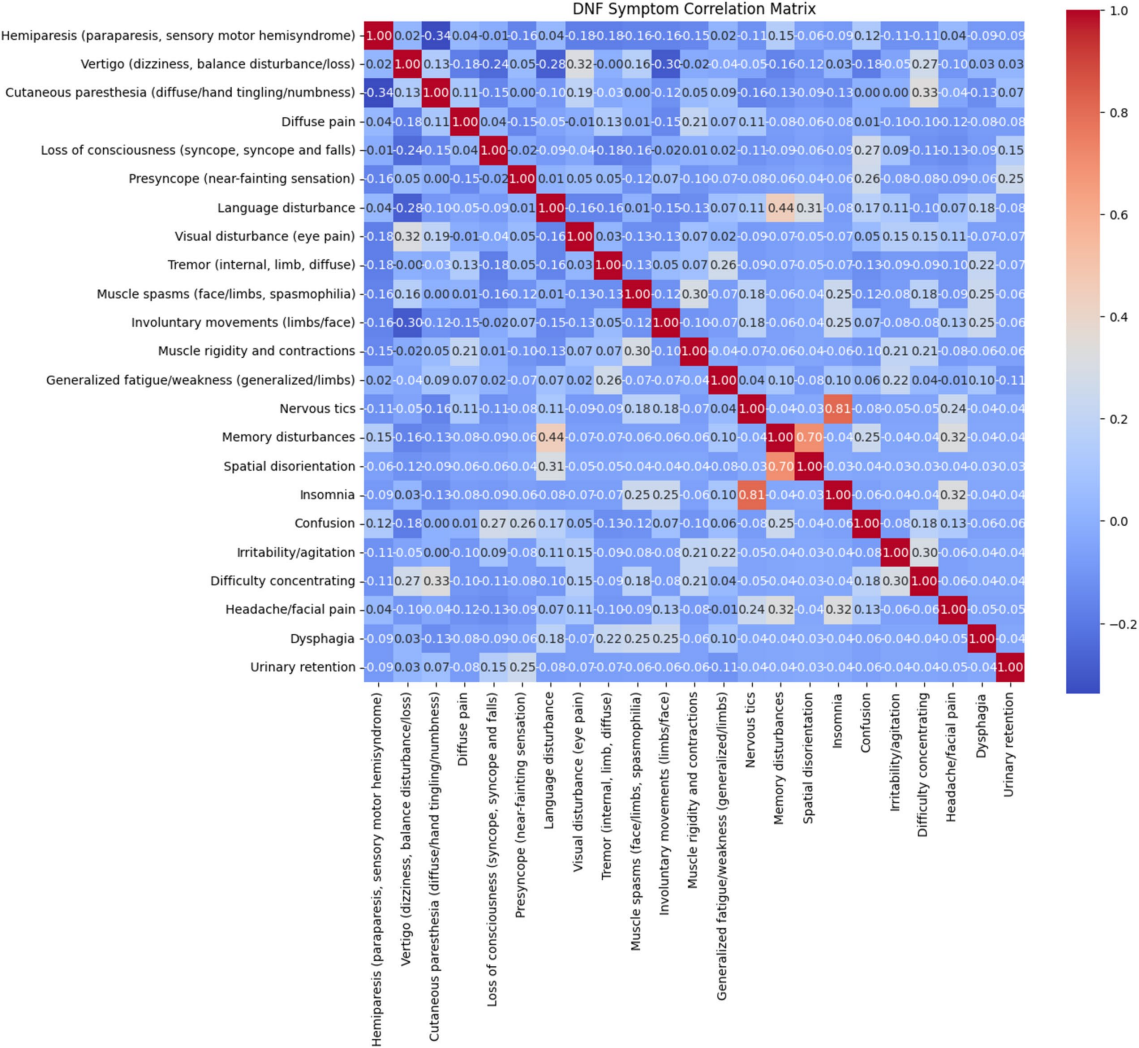
Matrix correlation between FND symptoms reported from the patients in the emergency room.

The Welsh test for the FEAR factor (one-tailed *p* = 0.04) demonstrates statistical significance at the 5% level, indicating that patients with functional neurological disorders have significantly higher mean fear scores than the female normative population (Table 3; Giacolini et al., 2017). This result could have some implications for managing and treating such patients, emphasizing the potential need for a specific focus on fear management in therapies and support strategies (Figure 3).

**Table 3:** Descriptive statistics with Welch t-values.

Variables	M_p_	SD_p_^2^	M_s_	SD_s_^2^	*t*	*p*
SEEKING	25.53	13.32	25.09	44.08	0.31	0.37
PLAY	23.86	21.16	22.13	41.85	1.25	0.11
CARE	27.67	22.18	28.96	25.50	−1.17	0.12
FEAR	23.82	31.36	26.13	36.57	−1.76	0.04
ANGER	21.94	26.42	19.65	78.33	1.22	0.11
SADNESS	24.32	19.89	26.30	47.77	−1.35	0.09

M_p_ is the normative sample means (Pascazio et al., 2015); SD_p_^2^ standard deviation of the female normative sample (Pascazio et al., 2015); M_s_ is the mean scores of the female fnd’s sample group; SD_s_^2^ is the standard deviation of our female fnd sample; *t* is referred to the Welch t-values; *p* is referred to the *p*-values.

The repeated measures ANOVA revealed a significant effect of time on SANDNESS scores [*F*(2,52) *p* = 0.009). This indicates a statistically significant decrease in Sadness scores across the three-time points (*W* = 0.844 *p* = 0.120).

**Figure 4:**
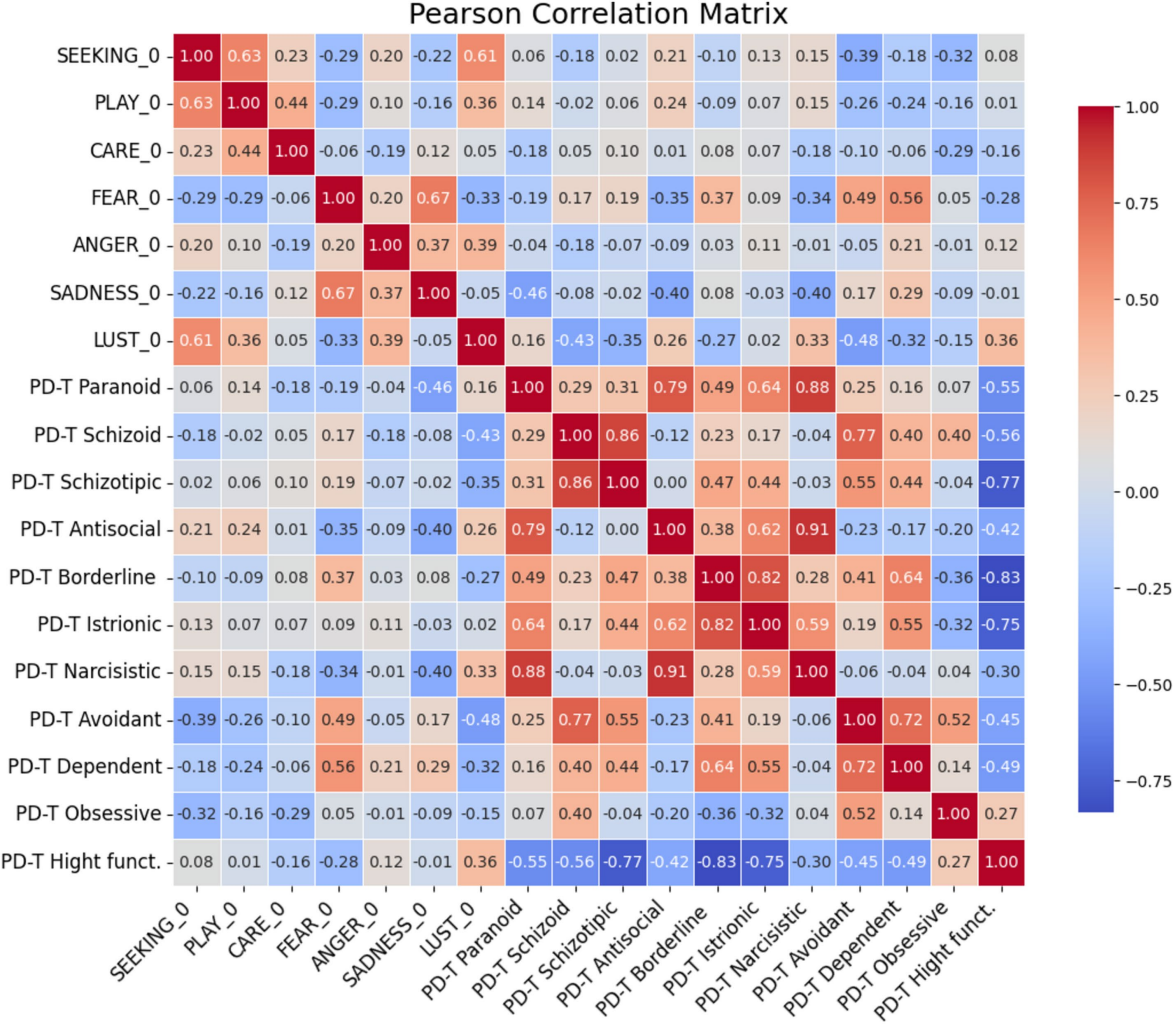
Pearson’s correlation matrix between ANPS variables and SWAP-200’S PD-T factors.

The matrix correlation between ANPS variables and PD-T factors (Figure 4) present the following significant correlations between the two scale:

1. SEEKING and PD-T Avoidant (Pearson *r* = −0.39 *p* ≤ 0.05):
  - There is a significant negative correlation between seeking behaviours (SEEKING) and avoidant personality traits (PD-T Avoidant) suggesting that individuals who score higher in seeking behaviours are less likely to exhibit avoidant traits.
2. FEAR and PD-T Avoidant (*r* = 0.49 *p* ≤ 0.01):
  - A significant positive correlation was found between fear (FEAR) and avoidant personality traits (PD-T Avoidant). Individuals with higher fear scores tend to have stronger avoidant traits, which aligns with the avoidance of anxiety-inducing situations.
3. FEAR and PD-T Dependent (*r* = 0.56 *p* ≤ 0.01):
  - There is a strong positive correlation between fear (FEAR) and dependent personality traits (PD-T Dependent), indicating that individuals with higher fear levels may also exhibit stronger dependency traits, relying on others for support.
4. SADNESS PD-T Paranoid (*r* = −0.46 *p* ≤ 0.05):
  - A significant negative correlation between sadness (SADNESS) and paranoid traits (PD-T Paranoid) suggests that individuals with higher levels of sadness may also display higher levels of suspicion and distrust.
5. SADNESS and PD-T Antisocial (*r* = −0.40 *p* ≤ 0.05):
  - There is a significant negative correlation between sadness (SADNESS) and antisocial traits (PD-T Antisocial). This relationship might indicate that sadness could be related to tendencies towards antisocial behaviors.
6. SADNESS PD-T Narcissistic (*r* = −0.40 *p* ≤ 0.05):
  - A significant correlation between sadness (SADNESS) and narcissistic traits (PD-T Narcissistic) may suggest a complex relationship where feelings of sadness coexist with narcissistic tendencies, potentially due to underlying self-esteem issues.
7. LUST PD-T Schizoid (*r* = −0.43 *p* ≤ 0.05):
  - A significant negative correlation between lust (LUST) and schizoid traits (PD-T Schizoid) indicates that individuals with higher levels of sexual desire may be less likely to exhibit the emotional coldness and detachment characteristic of schizoid personality.
8. LUST PD-T Avoidant (Pearson *r* = −0.48 *p* = 0.01):
  - There is a significant correlation between lust (LUST) and avoidant traits (PD-T Avoidant), suggesting that individuals with higher levels of sexual desire might also show tendencies to avoid social situations, possibly due to fear of rejection.

**Figure 5:**
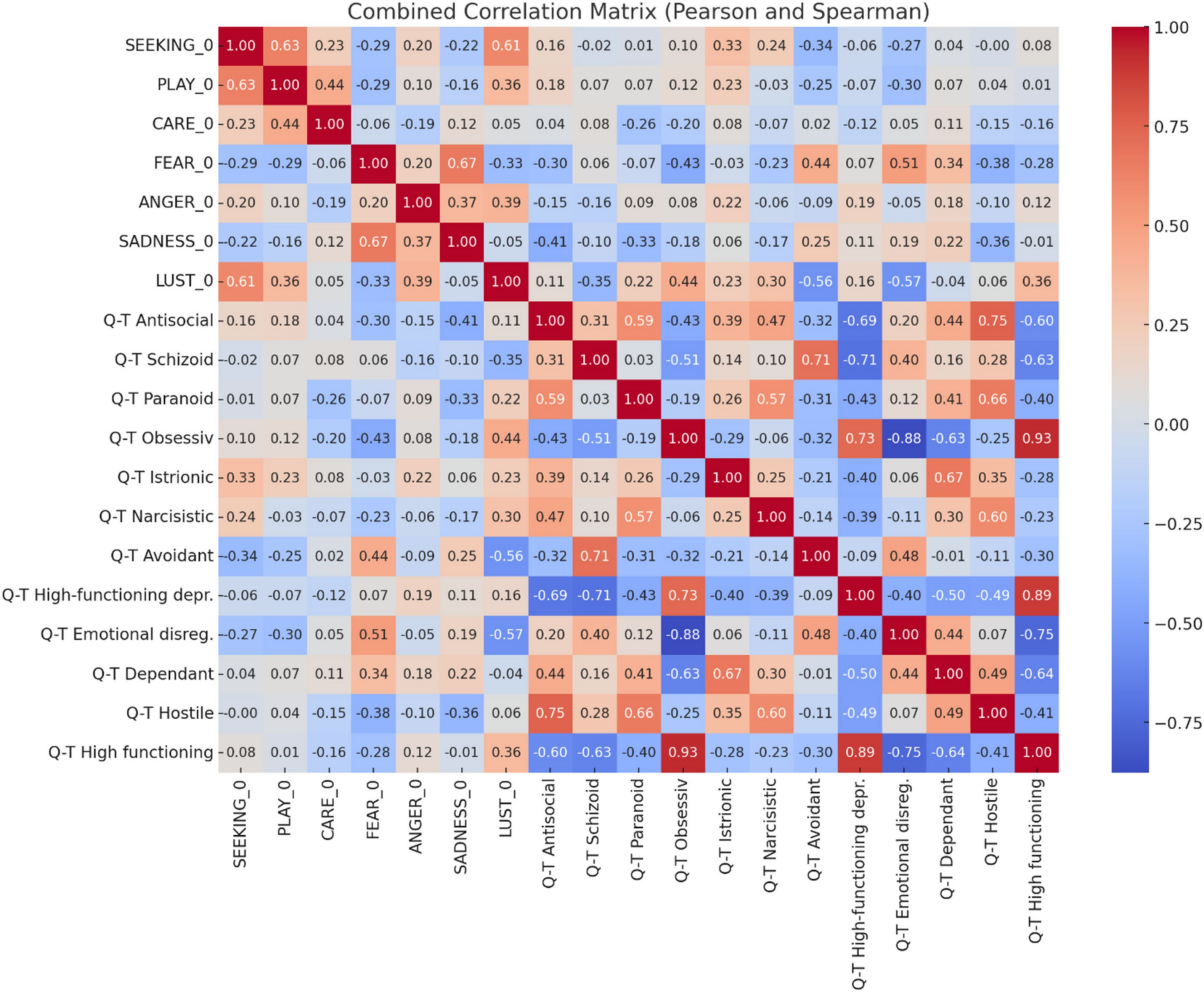
Spearman’s correlation matrix between ANPS variables and SWAP-200’s Q-T factors.

In the matching between ANPS variables and Q-T factors (Figure 5) the analysis identified several significant correlations:

1. SEEKING vs. PLAY: (*r* = 0.63 *p* ≤ 0.01).
  - Higher seeking behaviour is strongly associated with higher playfulness.
2. SEEKING vs. LUST: (*r* = 0.61 *p* ≤ 0.01).
  - A strong positive correlation indicates that seeking behaviour is associated with higher levels of lust.
3. PLAY vs. CARE: (*r* = 0.44 *p* ≤ 0.01).
  - There is a moderate positive relationship between playfulness and caring behaviour.
4. FEAR vs. SADNESS: (*r* = 0.67 *p* ≤ 0.01).
  - Individuals with higher levels of fear also tend to experience higher levels of sadness.
5. FEAR_0 vs. Q-T Avoidant: (*r* = 0.44 *p* ≤ 0.03).
  - Fear is positively correlated with avoidant traits.
6. FEAR_0 vs. Q-T Hostile: (*r* = −0.38 *p* = 0.05).
  - Higher fear levels are associated with lower hostility traits.
7. Q-T Obsessive vs. Q-T Hostile: (*r* = −0.43 *p* ≤ 0.05).
  - There is a moderate negative correlation between obsessive traits and hostility.

## Discussion

The results of our study provide an insight into the demographic, clinical, and neurological characteristics of FND patients. The high acceptance rate of psychological support across all groups is encouraging, suggesting that patients are open to integrative approaches that combine neurological and psychological care. This is particularly relevant given the complex interplay between psychological factors and functional neurological disorders (Table 4).

**Table 4:** Repeated measures ANOVA.

Effect within subjects					
	Sum of square	DF	Quadratic mean	*F*	*p*
Time	80.7	2	40.35	5.20	0.009
Residual	403.3	52	7.76		
Type 3 Sum of Squares					

Effect between subjects					
	Sum of square	DF	Quadratic mean	*F*	*p*
Residual	2,765	26	106		
Type 3 Sum of Squares					

Sphericity test				
	W di Mauchly	*p*	Greenhouse–Geisser *ε*	Huynh-Feldt *ε*
Time	0.844	0.120	0.865	0.921

The prevalence of psychiatric disorders varied among the groups, with Anxiety being most common in the Movement Disorders group, which aligns with previous literature indicating a strong link between anxiety and movement disorders (Pascazio et al., 2015). Panic attacks were also more frequently reported in this group, further emphasizing the need for comprehensive mental health assessments in patients presenting with movement-related symptoms.

The findings highlight significant emotional alterations, particularly in the FEAR and PANIC systems, which are neurobiological mechanisms essential for survival (Panksepp, 2010; Panksepp, 2011a; Panksepp, 1998). The FEAR system relates to protecting personal integrity, while the PANIC system reflects the need for proximity and connection. These systems are linked to chronic anxiety, separation anxiety, and fear (Panksepp, 2005a; Panksepp, 2005b; Panksepp, 2010; Panksepp, 2011a; Panksepp, 2011b).

Patients in our study demonstrated withdrawal from daily activities due to physical and psychological symptoms, including anxiety and irritability. The tests revealed abnormal activation of these systems, particularly in correlation with Narcissistic, Avoidant, and Dependent traits. These patients, focused on self-esteem and self-efficacy, simultaneously experience deep anxiety over losing significant others, underlining a conflict between autonomy and attachment. Notably, the PANIC system’s activation decreased after psychotherapy, reflecting positive changes in attachment dynamics.

Trust issues and fear of abandonment, manifested through defence mechanisms like projection and idealization (Clarkin et al., 1999; Sandler, 1960), also emerged, confirming earlier findings about patients with functional neurological disorders. Our psychotherapeutic approach, grounded in Transference Focused Psychotherapy (Clarkin et al., 1999), facilitated the analysis of patients’ internal object representations, highlighting how physical symptoms might serve as a maladaptive response to separation anxiety, seeking care from significant others. This aligns with earlier research (Stone et al., 2020; Edwards, 2021; Bennett et al., 2021).

These results suggest that symptoms in FND patients may stem from unresolved emotional conflicts, where bodily sensations become misinterpreted as signals of distress during crises. This emotional distress, when chronic, is deeply embedded in neural networks, as described in earlier studies (Solms and Friston, 2018; Stone et al., 2020), making early diagnosis and intervention crucial. Neurobiological changes in the limbic-motor axis of FND patients, related to emotion and movement management, further support this hypothesis (Aybek et al., 2015; Aybek et al., 2014; Liang et al., 2021).

Emotional factors should be considered primary indicators of underlying psychological suffering in FND patients, shaped by complex genetic, family, and environmental factors (Aybek et al., 2015; Davis and Panksepp, 2011; Espay et al., 2018). Recognizing these signals could lead to more effective diagnosis and treatment strategies.

The significant reduction in SADNESS scores after treatment, as shown by repeated measures ANOVA, highlights the intervention’s success in reducing anxiety linked to separation and loss. This improvement is likely due to the support of a professional sensitive to FNDs, who addresses the patient’s intense emotions of frustration and worry. Overall patients report an improvement in the symptomatology of functional symptoms through psychotherapy. However, it is important to note that these aspects are qualitative and have not been tested nor measured. Nonetheless, since ANPS is a self-reported test, the decrease in SADNESS might be correlated to symptoms’ improvements. Further studies with bigger randomized samples and longer trials are needed to clarify this possibility as well as to compare with current wider studies (Goldstein et al., 2020).

The correlations identified between ANPS and PD-T factors, as well as between ANPS and Q-T factors, provide essential insights into the psychological profiles of the patients. The significant correlations between FEAR and PD-T Avoidant, as well as between SADNESS and various PD-T traits, suggest that these emotional states are closely linked with specific personality characteristics. These findings could have important implications for developing targeted interventions that address functional neurological disorders’ emotional and personality dimensions.

Our study highlights the complex and multifaceted nature of functional neurological disorders, emphasizing the importance of a holistic approach to diagnosis and treatment that integrates both neurological and psychological care. Future research should build on these findings by incorporating larger sample sizes, control groups, and longitudinal designs to further elucidate the relationships between clinical, psychological, and neurological factors in these conditions.

## Highlights and limitation

The current study’s sample size of 58 subjects needs to be revised to reliably perform multinomial logistic regression analyses with the numerous predictive variables we considered. Given the complexity of the model and the multiple categories within the dependent variable, a larger sample is necessary to achieve stable and interpretable results.

Furthermore, the correlation analysis could be utterly refined considering FND subcategories (PNES, movement disorder, stroke mimic). Again, sample size did not allow this possibility, which shall be explored in further studies.

The dual use of Spearman and Pearson correlations in these analyses provided a balanced perspective. Spearman’s method was essential for capturing non-linear, monotonic relationships, especially in the context of psychological traits, while Pearson’s correlation offered precise insights into linear associations. Together, they allowed for a comprehensive understanding of the complex relationships between ANPS, PD-T, and Q-T factors, with each method addressing the limitations of the other. This combined approach ensures that the analysis captures a broad spectrum of potential relationships, enhancing the robustness of the findings.

## Conclusion

In conclusion, the repeated measures ANOVA provides strong evidence that the psychological intervention significantly reduced SADNESS levels over time, with the effect persisting beyond the immediate conclusion of the treatment. The underlying assumptions were adequately met, reinforcing the validity of these findings. These results contribute valuable insights into the longitudinal effectiveness of psychological therapies for emotional distress.

The findings from the correlation analyses between ANPS, PD-T, and Q-T factors, which derive from SWAP-200, provide important insights into the psychological profiles of these patients. However, these results must be discussed critically within the specific context of FND to understand their implications fully.

These findings emphasize the importance of a holistic approach to treatment that addresses both the psychological and neurological components of FND. However, the specific nature of this patient group means that these results should be interpreted with caution when considering broader applications. Further research is needed to explore how these findings might inform treatment strategies, improve FND patients’ outcomes, and determine whether similar patterns are observed in other clinical populations.

## Data Availability

The raw data supporting the conclusions of this article will be made available by the authors without undue reservation.
